# Alcohol use and risk of vehicle accidents: cross-sectional study in the city of São Paulo, Brazil

**DOI:** 10.1590/1516-3180.2019.0470.R1.27012020

**Published:** 2020-06-01

**Authors:** Janaina Barbosa de Oliveira, Florence Kerr-Corrêa, Ícaro Caresia Lopes, Walter Vitti, Hélio Rubens de Carvalho Nunes, Maria Cristina Pereira Lima

**Affiliations:** I PhD. Psychologist, Centro de Estudos da Educação e da Saúde/Centro Especializado em Reabilitação (CEES/CER II), Universidade Estadual Paulista (UNESP), Marília (SP), Brazil.; II PhD. Psychiatrist and Full Professor, Department of Neurology, Psychology and Psychiatry, Faculdade de Medicina de Botucatu (FMB), Universidade Estadual Paulista (UNESP), Botucatu (SP), Brazil.; III MSc. Psychologist, Department of Public Health, Faculdade de Medicina de Botucatu (FMB), Universidade Estadual Paulista (UNESP), Botucatu (SP), Brazil.; IV PhD. Physician and Professor, Department of Public Health, Faculdade de Medicina de Botucatu (FMB), Universidade Estadual Paulista (UNESP), Botucatu (SP), Brazil.; V PhD. Statistician and Methods and Statistics Consultant, Department of Public Health, Faculdade de Medicina de Botucatu (FMB), Universidade Estadual Paulista (UNESP), Botucatu (SP), Brazil.; VI PhD. Psychiatrist and Associate Professor, Department of Neurology, Psychology and Psychiatry, Faculdade de Medicina de Botucatu (FMB), Universidade Estadual Paulista (UNESP), Botucatu (SP), Brazil.

**Keywords:** Alcohol drinking. Accidents, traffic. Risk factors. Cross-sectional studies. Mental health. Psychopathology. Epidemiology, Alcohol and other drugs, Neuroscience, Gender and health

## Abstract

**BACKGROUND:**

Harm to other people caused by an individual under the influence of alcohol (UIA) can occur in a variety of relationship situations between the drinker and these other people.

**OBJECTIVES:**

To estimate the prevalence of the risk of vehicle accidents (RVA) involving people who are UIA, according to sociodemographic variables, respondent alcohol use and gender.

**DESIGN AND SETTING:**

Cross-sectional study, in which a household survey was carried out on a cluster-stratified representative sample of urban residents in the city of São Paulo.

**METHODS:**

The final sample was composed of 1,155 subjects aged 18-59 years, who were interviewed using the GENACIS Harm-to-Others questionnaire. Individuals were defined as having been harmed if an affirmative response was given to at least one of the questions that refers to RVA involving people who had been UIA in the last twelve months. Post-stratification weights were calculated to adjust for the study design and for no response. Since the outcome was binary, logistic regression was used in multivariable analysis.

**RESULTS:**

The final response rate was 58.6%. The overall prevalence of RVA was 13.6% (95% confidence interval, CI 11.0-16.7): 16.6% among men and 10.8% among women. After the logistic regression, age remained as a protective factor (odds ratio, OR 0.95) and binge drinking as a risk factor (OR 2.03).

**CONCLUSION:**

This study showed that binge drinking was associated with RVA.

## INTRODUCTION

Harm to other people caused by individuals who are under the influence of alcohol (UIA) can happen in many ways, to different degrees and to people with different relationships with the drinker. Harm to other people due to alcohol consumption can include intentional or unintentional injury and can affect a whole group or single individuals, who may be spouses, relatives, friends, non-relatives living in the same home, co-workers or even strangers.^1^^,^^2^^,^^3^

Studies have shown that traffic accidents and alcohol consumption are directly related, thereby causing serious consequences to individuals, families and the community.^4^^,^^5^^,^^6^^,^^7^^,^^8^ Brazil is one of the countries with the highest rates of traffic accidents relating to alcohol consumption, with huge social costs.^4^^,^^5^^,^^6^^,^^7^^,^^8^ A recent review by Araújo et al.^9^ assessed several studies on traffic accidents and alcohol use in Brazil, and showed that there was high prevalence of alcohol use and an association between this and the severity of injuries. A Brazilian survey conducted in 2012 indicated that the prevalence of individuals who reported driving after consuming alcohol in the last year was 27.3% among men and 7.1% among women.^10^

Epidemiological studies evaluating alcohol use relating to fatalities in the state of São Paulo, the Federal District and the city of Porto Alegre found alcohol in the blood in 45%, 43% and 32% of the subjects, respectively. Among non-fatal accident victims treated in trauma and emergency care centers in Uberlandia, state of Minas Gerais, 31.8% of these patients tested positive for blood alcohol content, and they more frequently required hospitalization (70.4% versus 37.9%; P < 0.05).^11^^,^^12^

A study carried out in the city of Rio de Janeiro showed that 42.5% of fatal traffic accidents involved individuals with some level of blood alcohol concentration, among which 66.2% had levels between 0.6 and 2.0 g/L of alcohol in the blood. Four to eight doses of alcohol, on average, in the last hours before the accident may indicate excessive alcohol consumption. However, this study in Rio de Janeiro found that the frequency of blood alcohol tests was low: only 34.8% of all victims of traffic accidents underwent the test. This will have contributed to underreporting of the true numbers of alcohol-related accidents in traffic.^13^

A cross-sectional study developed in the emergency services of Brazilian state capitals and in the Federal District showed that among the cases from traffic accidents that were attended, especially those involving motorcycles, the proportion in which alcohol consumption was a factor ranged from 5.8% in Rio de Janeiro to 28.6% in Teresina (state of Piauí).^14^ The city of São Paulo, the setting of the present study, is situated in the southeastern region of Brazil, which is the region of this country with highest lifetime rate of alcohol use (80.4%).^15^

In 2008, the Brazilian Congress approved law no. 11.705/2008, which reduced to zero the level of alcohol in blood that was permitted for drivers in this country.

The current study is part of GENAHTO, an acronym for Gender and Alcohol’s Harm to Others.^16^ “GEN” signifies the importance of gender in studying the harm that alcohol causes to other people, along with the project’s continuity with the GENACIS Project (Gender, Alcohol and Culture: an International Study). In the latter, several cross-sectional studies carried out in different countries had the aim of evaluating the patterns of alcohol use between men and women in different contexts and cultures, from a gender perspective.^3^^,^^17^

GENAHTO analyses are guided by a conceptual model in which the harm that alcohol causes to other people is examined within a nested multi-level set of potential influences. The central aspect of these analyses is the characteristics of individuals experiencing and causing this harm, such as gender and drinking patterns. These characteristics are entrenched in the individual’s role relationships, social groups and drinking contexts and provide extensive data on harms due to others’ drinking, from the victim’s perspective.^16^ GENAHTO aims to contribute towards elaboration of preventive and interventional measures for problems relating to use of alcohol by men and women. Through this contribution, measures that are sensitive to gender issues and policies regarding alcohol use can be developed. This research is carried out through a standardized questionnaire, with adaptation to different cultures.

## OBJECTIVE

The aim of the present study was to estimate the prevalence of the risk of vehicle accidents (RVA) involving people who were UIA, according to sociodemographic characteristics and respondent alcohol use.

## METHODS

### Sample

This study comprised a cross-sectional survey of the general population that was carried out on a cluster-stratified representative sample of urban residents of the city of São Paulo. The participants were aged 18 to 59 years. The sample was probabilistic.

Stratified sampling procedures were used in three stages: census tract, household and resident. Considering that only one resident in each household would be interviewed, 1,250 households were included in the sample. These households were distributed in 50 census tracts. In each census tract, 25 households were surveyed and, in each household, a single resident. To select this resident, we used the procedure proposed by Kish.^18^ Considering that empty dwellings would be found and that the refusal rate among the residents was likely to be 20%, 1,600 households were drawn, i.e. 32 in each census tract. If the home was found to be unoccupied, a maximum of two further visits were made, to try to find a resident. The interviews were conducted between September 2014 and January 2015. The percentage of the households that received the interviewers was 71.0%, but 17.5% of the households refused to participate, which resulted in a final response rate of 58.6%.

### Setting

The study site was the city of São Paulo, which is the capital of the state of São Paulo. This is the largest and the most populated city in Brazil and in the southern hemisphere. The Brazilian Institute for Geography and Statistics calculated the population of this municipality as 11,253,503 inhabitants in 2010. In this city, there were over 8.5 million registered motor vehicles in 2017; in 2016, there were 16,052 accidents, with 19,235 victims, including both fatal and non-fatal cases.^19^^,^^20^

### Dependent variable: risk of vehicle accidents (RVA)

RVA was the dependent variable in this study. Individuals were defined as having been harmed if an affirmative response was given to at least one of the questions referring to vehicle accidents that involved people who were UIA, in the last twelve months. The dependent variable was constructed through the following questions:

- Have you ever been a passenger of a driver who had drunk a lot?
- Have you had a car accident (or other motor vehicle accident) because of someone else’s drinking?
- Did you feel at risk in the car when someone else was driving, because of how much the person had drunk?
- Were you injured in a car accident because of how much someone else had drunk?

### Independent variables

The instrument used for data-gathering was the GENACIS Harm-to-Others questionnaire (available upon request):

• Sociodemographic variables
All sociodemographic characteristics, including the variables of gender, age, color, income and education were included in this study and were assumed to be independent for the analysis.
• Respondent alcohol use
The respondents were questioned about their alcohol consumption. They were classified as abstinent (had never made use of alcohol in their lifetime), former drinker (had not used alcohol in the last 12 months) or current drinker (had had alcohol intake in the last 12 months. The drinkers reported their consumption in terms of quantity and frequency on a typical day. Binge drinking was also investigated, and this was considered to consist of consumption of four or more drinks for women and five or more drinks for men on one occasion in the last 12 months. For the analysis, the number of doses on a typical day, any occurrences of binge drinking and the score from the alcohol use disorders identification test (AUDIT) were included, for current drinkers only.
• AUDIT
The AUDIT^21^ is an instrument with 10 items that was designed to identify the patterns of alcohol use. It has been validated for use in Brazil.^22^ When respondents get a score of eight points or more, their consumption is considered to be at a problematic level of use. Although this test includes questions that identify binge drinking, its aim is to identify subjects who already present a problematic pattern of use, as defined by this instrument.

### Analytical model

Associations between the outcome and independent variables were evaluated using the Rao-Scott test.^23^ Each observation was weighted accordingly to compensate for the effects of delineation and of no response. Firstly, we ran bivariate analyses, to select eligible variables for inclusion in multivariable analysis. We used multivariable logistic regression analysis, with backward stepwise variable selection.^24^ All the variables that showed associations with outcome presenting P ≤ 0.25 were included in the models.

The results from these analyses are presented in the form of odds ratios (OR), using 95% confidence intervals (CI). Only variables for which the associations presented P < 0.05 were retained in the final model. We did separate analyses for men and women. The data were analyzed using the Stata 12.0 statistical software (StataCorp LP, College Station, TX, USA).

### Ethics

The proposal for this study was approved by the Research Ethics Committee of the Medical School, Universidade Estadual Paulista (UNESP), on November 5, 2012 (protocol no. 4410-2012), and was supported by the Research Support Foundation of the State of São Paulo Research Program for the National Health System (Fundação de Amparo à Pesquisa do Estado de São Paulo-Programa de Pesquisa para o SUS, FAPESP-PPSUS) (grant number 2012/51237-8).

## RESULTS

The final sample was composed of 1,155 subjects, among whom there were 648 women (52.3%) and 507 men (43.7%). The final response rate was 58.6%. The prevalence of RVA was 13.6% (95% CI 11.0-16.7%) and it was significantly higher among men (P = 0.01): 16.6% among men (95% CI 12.0-21.2%) and 10.8% among women (95% CI 8.1-13.5%).

As mentioned earlier, bivariate analysis was run for each gender. Regarding demographic variables (Table 1), exposure to RVA due to alcohol use by other people was significantly higher among the following groups of men: those who were younger (29.0%), single (24.4%), with schooling levels ranging from incomplete high school to incomplete college/university (20.3%), with no children (22.9%) and students (46.6%). Among women, only the education level of the person responsible for the family income was significant (P = 0.01). RVA was most prevalent among individuals whose schooling level ranged from incomplete high school to incomplete college/university (14.0%).

**Table 1.** Risk of vehicle accidents involving people who were under the influence of alcohol in the last 12 months, according to sociodemographic characteristics (n = 1,155), city of São Paulo, 2014

Sociodemographic characteristics	Risk of vehicle accidents Males (n = 507)			Risk of vehicle accidents Females (n = 648)		
	n	%	P*	n	%	P*
Age			< 0.001			0.72
< 30	41	29.0		20	12.4	
30 to 49	37	12.9		33	9.9	
≥ 50	4	4.0		12	10.6	
Color			0.83			0.68
White	33	17.1		34	11.4	
Nonwhite	49	16.2		31	10.2	
Marital status			0.004			0.25
Married	28	10.5		28	9.1	
Widowed/divorced/separated	6	14.4		12	9.7	
Single	48	24.4		25	13.5	
Education			0.04			0.20
Only elementary school	4	8.7		11	11.0	
Incomplete high school to incomplete college/university	55	20.3		40	12.7	
Completed college/university or more	23	12.8		14	6.6	
Children			0.01			0.79
Yes	36	11.2		46	10.6	
No	46	22.9		19	11.2	
Current work status			0.03			0.62
Employee or self-employed	66	15.5		44	11.2	
Retired	1	7.1		2	15.9	
Unemployed	11	21.2		11	12.1	
Student	4	46.6		2	14.2	
Housekeeper	0	0		6	5.9	
Monthly family income			0.82			0.77
0 to 3 minimum wages	47	16.5		45	10.9	
> 3 minimum wages	33	15.6		16	10.1	
Education level of the person responsible for family income			0.45			0.01
Only elementary school	26	12.5		12	5.1	
Incomplete high school to incomplete college/university	40	18.1		42	14.0	
Completed college/university or more	13	19.3		11	11.6	

^*^Rao-Scott test. Percentages were calculated considering weights for non-response and for study design.

As shown in Table 2, among men, occurrences of vehicle accidents involving people who were UIA in the last 12 months were associated with being a current drinker (19.2%). It is noteworthy that, for this variable, a dose-response effect could be seen among lifetime abstainers, former drinkers and current drinkers. In addition, reporting occurrences of consumption of five or more alcoholic beverages on a single occasion in the last 12 months (26.2%), consuming five or six drinks on a typical day (30.3%) and AUDIT scores of eight or more points (29.2%) were associated with RVA.

**Table 2.** Risk of vehicle accidents involving people who were under the influence of alcohol in the last 12 months according to alcohol consumption and according to gender, city of São Paulo, 2014

Risk factor	Risk of vehicle accidents Males (n = 507)			Risk of vehicle accidents Females (n = 648)		
	n	%	P	n	%	P
Alcohol consumption			0.008			
Abstinent	1	1.5		11	5.7	0.0001
Former drinker	13	14.6		10	5.6	
Current drinker	68	19.2		44	16.3	
Frequency of alcohol consumption in the last 12 months			0.35			
Never	14	10.7		21	5.6	0.001
Monthly or less	13	19.8		15	14.9	
2 to 4 times per month	46	18.6		28	17.9	
2 to 3 times per week	3	17.9		0	0	
4 or more times a week	6	26.0		1	15.6	
Binge drinking^a,b^			0.0002			
No	15	9.7		18	13.6	0.17
Yes	53	26.2		26	19.8	
Drinks on a typical day^a^			0.03			
1 to 4	34	15.0		29	14.1	0.10
5 to 6	13	30.3		7	27.6	
7 to 9	9	29.3		3	39.9	
≥ 10	12	28.7		5	31.1	
AUDIT score ≥ 8^c^			0.0002			
No	25	12.7		27	14.4	0.10
Yes	43	29.2		17	25.2	

AUDIT = alcohol use disorders identification test.

Percentages were calculated considering weights for non-response and for study design. ^a^Information only among drinkers; ^b^Four or more drinks for women and five or more drinks for men on one occasion in the last 12 months; ^c^Alcohol use identification test.

Among women, occurrences of vehicle accidents involving people who were UIA in the last 12 months were associated with being a current drinker (16.3%) and high frequency of alcohol consumption in the last 12 months (P = 0.001).

For the multivariable analysis, it was decided to build a single model for both genders together. All the variables selected were included in the model and they were retained if P > 0.05. In order to highlight the importance of binge drinking, we presented two models (Table 3). In the first model, only gender and age were included, and this showed the risk associated with male gender (OR = 1.64) and the protective effect of increasing age. In the second model, binge drinking was included and this nullified the risk associated with male gender. It was observed that the binge drinking variable was a risk factor (OR = 2.38) and that its introduction changes the OR values for gender, thus changing its significance. Also, the inclusion of binge drinking did not prejudice the role of age, which remained a protective factor.

**Table 3.** Logistic regression for risk of vehicle accidents involving people who were under the influence of alcohol in the last 12 months, city of São Paulo, 2014 (n = 1,155)

	Crude odds ratio	Confidence interval	Adjusted odds ratio	Confidence interval	P
Model 1					
Age	0.96	0.94-0.98	0.96	0.94-0.98	< 0.001
Gender					
Female	1	-	-	-	
Male	1.64	1.11-2.41	1.61	1.13-2.32	< 0.01
Model 2					
Age	0.96	0.94-0.98	0.95	0.93-0.98	< 0.001
Sex					
Female	1	-	-	-	
Male	1.64	1.11-2.41	1.11	0.75-1.66	0.60
Binge drinking^a^	2.38	1.53-3.71	2.03	1.26-3.25	0.004

^a^Four or more drinks for women and five or more drinks for men on one occasion in the last 12 months.

## DISCUSSION

Social harms from drinking are inherently interactional. For social harm to be recognized as occurring, there needs to be not only drinking behavior that is seen as problematic, but also a reaction by someone other than the drinker. The harm may occur to the drinker or to other people.^25^ Alcohol surveys have usually asked drinkers themselves about problems from their own drinking. However, it is important to investigate people who have been harmed by other people’s drinking.^26^

The total percentage of individuals who reported occurrences of RVA in the present study was 12.73%. Using data from the 2014 to 2015 United States National Alcohol Survey, Karriker-Jaffe et al.^27^ found that fewer than 1% of the respondents had been in traffic accidents caused by someone who had been drinking, in the last year.

The main outcome of the present study was that a significant association was found between higher prevalence of risks of vehicle accidents (RVA) and binge drinking, caused by other people’s consumption. In other words, when drinkers practice binge drinking, they put themselves at risk, regardless of whether or not they are the driver. A causal link has been shown between consumption of alcoholic beverages and occurrences of traffic accidents, considering the effects of alcohol on individuals’ perception, vision, reflexes, consciousness and behavior.^28^ A study conducted in all Brazilian state capitals and the Federal District showed that the percentage of the subjects who had consumed any amount of alcohol before driving was 5.3% in 2011 and 7.3% in 2016.^29^ The first National Household Survey on Alcohol Consumption Patterns, which was applied in 143 Brazilian cities between 2005 and 2006, indicated that 34.7% of motorists drank and drove in the last 12 months.^6^ Data from 2008 for the adult population of 27 cities showed that 1.5% of individuals reported having driven a motor vehicle after excessive alcohol consumption, on at least one occasion in the last 30 days.^7^ One study found that 41.2% of the 431 victims of fatal motor vehicle accidents over 16 years of age who were admitted to the Institute of Legal Medicine of the Federal District (IML-DF) presented a blood alcohol concentration above 0.6 g/l.^11^

In the present study, it was observed that age was a protective factor, such that RVA decreased with increasing age. The practice of driving after excessive alcohol consumption showed highest frequency in the 25 to 34-year age group (4.0% among men and 0.7% among women).^7^ Duailibi et al.^8^ studied drivers between 21 and 30 years old, on weekend nights in Diadema (SP) and observed that more than 20% of the drivers undergoing the breathalyzer had breath alcohol levels above 0.01 g/dl.

In Brazil, violent deaths, including homicides, traffic accidents and suicides represent about 70% of deaths among people between 15 and 24 years of age. This number places Brazil among the 10 countries where traffic accidents are responsible for more than 60% of deaths.^30^

Brazilian data from the telephone-survey surveillance system for risk factors and protective factors relating to chronic diseases (Sistema de Vigilância de Fatores de Risco e Proteção para Doenças Crônicas por Inquerito Telefônico, VIGITEL) for the period from February to December 2016 showed that the rate of alcohol abuse had increased from 15.7% to 19.1% over the preceding ten-year period.^29^ The second National Survey of Alcohol and Drugs, conducted among individuals aged 14 years and over, investigated consumption rates of five doses or more for men and four doses or more for women, on a single occasion within a period of up to 2 hours, in the last 12 months. The data showed prevalences of 45% in 2006 and 58% in 2012, i.e. increasing from 54% to 66% among men and from 34% to 48% among women.^10^

Although gender did not remain significant after multivariable analysis in the present study, data in the literature show that men are more likely than women to binge-drink and to drive after doing so. Data from a telephone survey among adults in the United States aged 18 years and over found that 75.1% of binge drinkers were men and that 49.7% consumed seven or more drinks during their most recent binge-drinking episode. In the gender-based assessment, men were more likely than women to drive after binge drinking (13.2% versus 8.1%), and men accounted for 82.9% of all recent binge drinking and driving episodes.^31^

In the 60^th^ World Health Assembly in 2007, binge drinking was shown to be responsible for 3.7% of deaths and was associated with 4.4% of diseases in the world. Thus, binge drinking is a public health problem and monitoring it is essential in order to find out about consumption patterns and ascertain which segments of the population are more vulnerable. These data are fundamental for formulating and funding public policies for health promotion and prevention of risky behavior.^32^

One possible limitation of the present study was the higher non-response rate among men and especially in census tracts with middle-to-high income. Higher non-response rates can be expected in large urban centers, especially in those with high incidence of violence and fear of this situation. However even developed countries can have low response rates: in a similar study carried out in Ireland, the response rate was 37%; while in Australia it was 38%.^16^ There is no consensus regarding the minimum response rate for population surveys, taking into account the characterization of non-responders, or regarding the extent to which this loss relates to the information sought by the study. Since the subject of RVA is a sensitive issue, we can assume that there is a risk minimization factor: probably the risk is greater than what has been found.

Our data differ from those of some other studies that indicated high abstinence rates in Brazil, especially in populations with lower levels of education.^33^^,^^34^ It is possible that this result is related to the questionnaire that was used. The GENACIS Harm-to-Others questionnaire assesses the frequency of alcohol consumption through detailed questions about different types of alcoholic beverages, with the aim of minimizing biases in memory, misrepresentation, false responses or non-acceptance, which are common in surveys on embarrassing or intimate issues.

Nonetheless, the figures obtained in the present study are close to the national data, which indicate that the prevalence of drinkers is around 50%.^10^ Also, as previously mentioned, the city of São Paulo is characterized by a peculiar situation, in that it is located in the region with highest lifetime rate of alcohol use (80.4%).^15^

 The strengths of this study, on the other hand, are that it was conducted over a short period, was stratified according to neighborhoods and had a representative sample of residents of the city of São Paulo, between 18 and 59 years of age. Thus, the results can be generalized to other large urban centers and probably do not reflect the patterns of rural areas of Brazil.

The problems arising from consumption of alcoholic drinks by drivers have been studied internationally in a broad manner. In Brazil, the risk of vehicle accidents associated with alcohol use can be considered to be a major public health problem, with a major impact on morbidity and mortality, especially in the young male population. Therefore, preventive interventions need to focus on these problems, for instance through promoting strict enforcement of drinking and driving laws to prevent this combination of behaviors.

## CONCLUSIONS

GENAHTO is the first project to assess the harm that alcohol use causes to other people in diverse societies.^16^ The current study aimed to estimate the prevalence of the risk of vehicle accidents involving people who were under the influence of alcohol, and it attempted to fill the gap regarding the lack of studies on people who are victims in accidents involving alcohol consumption by other people. It is important to highlight that we found that individuals who practiced binge drinking put themselves in RVA situations, regardless of whether they are the driver or not. Until now, this impact has not been considered in establishing public policies regarding alcohol consumption. This indicates that there is a need for further studies in this important area, and the results from the present study could be useful for guiding preventive strategies.
